# Molecular characterization of circulating tumor cells in lung cancer: moving beyond enumeration

**DOI:** 10.18632/oncotarget.22651

**Published:** 2017-11-23

**Authors:** Lei Wang, Coraline Dumenil, Catherine Julié, Violaine Giraud, Jennifer Dumoulin, Sylvie Labrune, Thierry Chinet, Jean-François Emile, Biao He, Etienne Giroux Leprieur

**Affiliations:** ^1^ Department of Thoracic Surgery, Fourth Hospital of Hebei Medical University, Shijiazhuang, Hebei, China; ^2^ Thoracic Oncology Program, Department of Surgery, Helen Diller Family Comprehensive Cancer Center, University of California, San Francisco, CA, USA; ^3^ Department of Respiratory Diseases and Thoracic Oncology, APHP – Ambroise Pare Hospital, Boulogne-Billancourt, France; ^4^ Department of Pathology, APHP – Ambroise Pare Hospital, Boulogne-Billancourt, France; ^5^ EA 4340 “Biomarqueurs en Cancérologie et Onco-Hématologie” UVSQ, Paris-Saclay University, Boulogne-Billancourt, France

**Keywords:** lung cancer, circulating tumor cells, prognosis, predictive marker, molecular diagnosis

## Abstract

Molecular characterization of tumor cells is a key step in the diagnosis and optimal treatment of lung cancer. However, analysis of tumor samples, often corresponding to small biopsies, can be difficult and does not accurately reflect tumor heterogeneity. Recent studies have shown that isolation of circulating tumor cells (CTCs) is feasible in non-small cell lung cancer patients, even at early disease stages. The amount of CTCs corresponds to the metastatic potential of the tumor and to patient prognosis. Moreover, molecular analyses, even at the single-cell level, can be performed on CTCs. This review describes the technologies currently available for detecting and capturing CTCs, the potential for downstream molecular diagnostics, and the clinical applications of CTCs isolated from lung cancer patients as screening, prognostic, and predictive tools. Main limitations of CTCs are also discussed.

## INTRODUCTION

Lung cancer is the main cause of cancer-related mortality worldwide [1]. Molecular screening techniques have allowed great advances in the treatment of non-small cell lung cancer (NSCLC) [2]. However, there is an urgent need to develop new tools to improve lung tumor cell characterization and understand the mechanisms underlying prognosis, treatment resistance, and tumor progression. Moreover, easy and reliable methods are still needed for lung cancer screening. Since a few years, the use and impact of liquid biopsy have raised as a surrogate marker of solid biopsy [3]. Liquid biopsy can be performed from many body fluids, but mainly from peripheral blood [4]. Along with circulating tumor DNA (ctDNA), circulating tumor cells (CTCs) are cells originating from the tumor site that migrate to the blood circulatory system and disseminate throughout the body; they have a putative role in metastasis formation [5, 6]. Their presence in patients with solid tumors at all disease stages is well established [7, 8], and they have the same cytomorphologic features as the original tumor [9]. CTC detection and isolation in lung cancer patients is technically difficult. However, molecular and functional analyses of lung cancer CTCs have great clinical potential. This review describes the different techniques currently available for detecting and isolating CTCs from lung cancer patients; the clinical impact on cancer screening, patient prognosis, and predicting the treatment response; and current data concerning the molecular analyses of CTCs.

## METHODS FOR CTC DETECTION AND ISOLATION

### Magnetic bead assays: EpCAM and CD45 depletion

CTCs can be directly detected or enriched using anti-epithelial cell surface molecule (EpCAM) antibody or indirectly enriched by removing other cells, for example using anti-CD45 antibody (specific for leukocytes). The Cellsearch system (Janssen Diagnostics) is designed to isolate CTCs from peripheral whole blood samples using EpCAM selection with magnetic beads. Cells are also stained with DAPI (nuclear staining), various cytokeratins (cytokeratins 8, 18, and 19), and CD45 for fluorescence microscopy analysis: CTCs are oval/round cytokeratin positive and CD45 negative cells with a visible nucleus (DAPI positive). Allard et al. validated this system in 964 patients with metastatic carcinomas, 199 patients with nonmalignant diseases, and 145 healthy controls [10]. In patients with metastatic lung cancer (*n*=168), the mean number of CTCs was 30 per 7.5 mL blood, and 20% of patients had ≥2 CTCs per 7.5 mL blood. Similar percentages were found in other studies [11–13]. Other systems aimed to improve the EpCAM-dependent assay, for example by using a microfluidic device with bidirectional flow and a sloped box to concentrate CTCs [14, 15] or by performing RT-PCR to measure expression of a panel of cancer genes after CTC enrichment [16]. The Cellsearch platform is currently approved by the US Food and Drug Administration for CTC isolation from blood samples from breast, colorectal and prostate cancer patients.

Depletion of CD45-positive cells with magnetic beads is another method of isolating CTCs. CD45 is a highly specific marker of leukocytes, the main nucleated cell population in the blood. Wu et al. tested this method in a cohort of 47 lung cancer patients (41 newly diagnosed patients and 6 with recurrent lung cancers), 13 tuberculosis patients and 18 healthy controls [17]. After CD45-positive cell depletion by cell sorting, cytokeratins 18 and 19 immunofluorescence assay was performed. Positive detection was defined as the presence of ≥2 CTCs per 7.5 mL blood. The overall detection rate with this cut-off was 85% in lung cancer patients versus 8% in tuberculosis patients and 0% in healthy patients. When stratified by tumor stage in newly diagnosed patients, the detection rate was 67% for stage I–II (mean, 5 CTCs per 7.5 mL), 72% for stage III (mean, 7 CTCs per 7.5 mL), and 50% for stage IV (mean, 7 CTCs per 7.5 mL). For recurrent cancer patients, the detection rate was 83% (mean, 11 CTCs per 7.5 mL). Another study with a similar design also reported high detection rates (73% for stage I–III cancer patients and 100% for stage IV cancer patients) [18].

Folate receptors are overexpressed in various solid tumors, including NSCLC [19]. A specific two-step method for CTC detection and isolation by recognizing surface folate receptor expression has been developed (CytoploRare, GenoSaber Biotech). The first step is CTC enrichment by leucocyte deletion (using CD45-coated immunomagnetic beads) and the second step is ligand-targeted PCR (LT-PCR) in which CTCs are labeled with the tumor-specific folic acid ligand conjugated to a synthesized oligonucleotide. The conjugated ligand is then annealed and extended onto the RT primer, before amplification and quantitative PCR. Using this method, the CTC detection rate is around 75% in lung cancer patients [20–22].

### Chip-based assays

CTCs can also be isolated using a microfluidic platform, called the “CTC-chip,” developed by Nagrath et al. In this assay, an anti-EpCAM antibody tethered to microposts captures EpCAM-positive cells as they pass through the device [23]. After sorting, CTCs were characterized by cytokeratin, CD45, and DAPI staining, as described for other assays. In a cohort of 116 cancer patients (55 metastatic lung cancer patients), the detection rate was 99% for NSCLC patients (with 5–1281 CTCs per ml) and 0% in 20 healthy controls. The captured cell purity (cytokeratin-positive cells/CD45-positive cells ratio) was 52% in NSCLC patients.

Many similar devices have been produced by different research groups based on the same principle (isolation of CTCs using mechanical pumps inducing inertial lift and drag forces). After passing through the chip, CTCs can be characterized by immunofluorescence staining and/or magnetic beads or nanoparticles coated with various antibodies (e.g. CD45, cytokeratins, EpCAM). The detection rate is often high (53–100%) in lung cancer samples [24–35]. Some devices include special membranes to improve CTC isolation, release, and viability [36–38], whereas other devices use centrifugal instead of mechanical force [39–42]; all have good detection rates.

Another device, called Apostream (ApoCell), is a microfluidic system for dielectrophoretic separation of CTCs from normal circulating cells. Enriched CTCs are then labeled with anti-folate receptor alpha antibody for immunofluorescence characterization. However, the CTC detection rate for this method is low: 36% for advanced lung adenocarcinoma patients (*n*=5/14) and 0% for lung squamous cell carcinoma patients (*n*=0/6) [43].

### Size-based assays

Due to their larger size compared with normal leukocytes, CTCs can be isolated by size-based filtration. This concept was first developed and validated in samples from hepatocellular carcinoma patients by Vona et al. in 2000 with the ISET system (Isolation by Size of Epithelial Tumor cells; Rarecells) [44]. ISET isolation enabled morphological and functional analyses of CTCs. Later, ISET isolation of CTCs was tested in a large cohort of 250 lung cancer patients and 59 healthy patients [45]. Based on their morphology, cells were classified as malignant (meeting at least four of the following criteria: anisonucleosis, nuclei >24 μm, irregular nuclei, high nuclear–cytoplasmic ratio, three-dimensional sheets), uncertain malignant cells (meeting 1–2 of the criteria) or benign cells (meeting none of the criteria). The detection rate was 41% for malignant cells and 6% for uncertain malignant cells; and none of the samples from healthy subject contained CTCs. Interestingly, intra-institution agreement in diagnosing malignant circulating cells (evaluation by three different pathologists) was very good (κ=1), but inter-institution agreement was poor (κ<0.40 for three different institutions compared with the referent institution).

The ScreenCell device uses a similar size-based approach for CTC isolation. For two different pathologists evaluating the presence of CTCs in blood samples from a cohort of 74 cancer patients (including 32 lung cancer patients), the individual detection rates were 53% and 57%, , but the rate of detection by both pathologists was only 43% [46] with a moderate κ value (0.54). Smaller series of lung cancer patients reported similar detection rates [47, 48].

A recent work from Yagi et al. described the development of an automated microcavity array (MCA) system for CTCs isolation [49]. This assay is based on the differences in size and deformability between tumor cells and normal blood cells. After isolation with MCA, cells were considered as CTCs if they were DAPI-positive, cytokeratin-positive, and CD45-negative under the fluorescence microscope. On 50 lung cancer patients and 10 healthy volunteers, sensitivity and specificity were 80 and 90%, respectively, with better performances than the Cellsearch system.

Other locally developed devices for size-based CTCs isolation include microcavity array [50, 51], fluid-assisted separation technology (FAST) [52], home-made membranes [53], and integrated microfluidic chip with membrane filter [54], all with good detection rates (69–100%).

### Other methods of CTC detection

Other assays have been tested for CTC detection. As cancer stem cells (CSCs) are the key factors for cancer spreading and metastases induction, Skirecki et al. hypothesized that CTCs had similar features to CSCs [55]. They performed flow cytometry analysis with CD133 (a CSC marker) and EpCAM double staining in both lung tumor tissues (*n*=7) and peripheral blood samples (*n*=41) from lung cancer patients. However, the CD133^+^/EpCAM^+^ cell detection rate was only 36% in peripheral blood compared with 86% in tumor tissues. Other research groups developed immunofluorescence assays for cell monolayers from blood samples after red blood cell lysis using various antibodies (e.g. against cytokeratins, CD45), with only limited preliminary results [56, 57].

*In vivo* assays for isolating CTCs directly from the peripheral veins of patient have also been described. Most use a wire coupled to an anti-EpCAM antibody directly inserted into the vein via a catheter, and have a detection rate of 58–100% [58, 59].

### CTCs and epithelial–mesenchymal transition

EpCAM is used as a marker for CTC isolation in several assays, for example in the Cellsearch device [10–13], based on the rational that all CTCs express epithelial markers. Thus, CTCs with a mesenchymal phenotype are not detected using this method. Epithelial–mesenchymal transition (EMT) is the process of conversion from an epithelial to a mesenchymal phenotype, resulting in expression of mesenchymal markers (e.g. vimentin) and loss of epithelial markers (e.g. E-cadherin and EpCAM). EMT is associated with cell migration and metastasis, and is a feature of CSCs [60, 61]. EMT markers are heterogeneously expressed in CTCs, and are mainly detectable in CTCs isolated by ISET compared with Cellsearch [62]. Most CTCs isolated by ISET frequently express both epithelial and mesenchymal makers, but some express only epithelial or mesenchyme markers [63, 64]. Bozzetti et al. showed that only 29% of CTCs isolated by ISET from 55 advanced squamous cell lung carcinoma patients were EpCAM positive, whereas 43% were vimentin positive [65]. De Wit et al. characterized EpCAM-negative cells discarded by the Cellsearch method by filtration and additional cytokeratins staining [66]. When discarded CTCs were included, the detection rate increased from 41% to 74%. Using this method, CTCs were detected in 33% of patients in whom no CTC was detected by Cellsearch. A recent study suggested that CTCs with EMT features were more frequent in EGFR-mutated NSCLC, compared to ALK-rearranged NSCLC and Kras-mutated NSCLC [67].

A direct comparison of CTC detection by ISET and Cellsearch has been performed in lung cancer patients. Krebs et al. showed a higher detection rate for ISET in a cohort of 40 patients with advanced NSCLC: 80%, for ISET versus 23% for Cellsearch [68]. Interestingly, most CTCs isolated with ISET did not express EpCAM. Clusters of CTCs (i.e. circulating tumor microemboli (CTM)) are associated with a greater propensity for metastasis compared with single CTC [69, 70]. CTM were observed in 43% patients when ISET was used for CTC isolation versus 0% with Cellsearch. Farace et al. tested concordance between the two methods in 60 lung cancer patients (20 with advanced NSCLC) [71]. Concordant results were obtained for only 20% of patients: 15% of patients had ≥3 CTCs per 7.5 mL with Cellsearch versus 60% with ISET. A median of 0 CTCs per 7.5 mL was obtained with for Cellsearch and 5 CTCs per 7.5 mL with ISET.

## CLINICAL IMPLICATION OF CTCS

### Prognostic impact of CTCs

Most studies on CTCs in lung cancer have shown an association between the presence of CTCs and poor prognosis, whatever detection method was used. The presence of CTCs seems to be associated with the TNM stage of the disease [11–13, 72–76]. CTC detection is consistently associated with lower progression-free survival (PFS) and overall survival (OS) [72, 74, 77–81].

For early-stage NSCLC treated by surgery, CTCs have been detected in the peripheral blood [82–89] and in pulmonary venous blood [90–96]. The CTC detection rate seemed to be higher in samples from the pulmonary vein compared with peripheral blood taken during surgery in the same patient [97]. However, whatever the sample site, the presence of CTCs is associated with poor outcome with shorter disease-free survival [82–89].

Huang et al. published a meta-analysis on the prognostic impact of CTCs in lung cancer patients that included 20 studies and 1576 patients [98]. CTCs were positively associated with tumor stage (odds ratio OR 1.95 (95% CI 1.08–3.54)) and nodal stage (OR 2.06 (95% CI 1.18–3.62)), and with poorer OS (relative risk RR 2.19 (95% CI 1.53–3.12)) and PFS (RR 2.14 (95% CI 1.36–3.38)). Another recent meta-analysis (8 studies and 453 patients) showed equivalent results [99].

### Predictive impact of CTCs

Changes in the CTC count before and during treatment correlate with treatment response and outcome, respectively. A decrease in CTC numbers during cytotoxic chemotherapy is associated with treatment response and longer PFS, whereas stable or increased CTC numbers during treatment is associated with disease progression and shorter PFS [72, 75, 78, 80, 100]. A meta-analysis by Wu et al. confirmed the predictive impact of CTC numbers [99]: the presence of CTCs at baseline was negatively associated with the disease control rate (RR 2.56; 95% CI 1.36–4.82) but not with the response rate (RR 1.07; 95% CI 0.75–1.53). However, persistence of CTCs after the 3rd cycle of chemotherapy correlated negatively with both the disease control rate (RR 9.39; 95% CI 2.81–31.39) and response rate (RR 1.85; 95% CI 1.04–3.30). Tarumi et al. showed in stage III NSCLC patients (*n*=9) after induction chemoradiotherapy that the absence of CTCs was associated with a complete pathological response (*p*=0.012) [101]. Changes in the CTC detection rate during chest radiotherapy also correlated with tumor response and PFS [102–104].

The same association was found for targeted therapies. Patients treated with pertuzumab (HER2 antibody) or erlotinib (EGFR tyrosine kinase inhibitor (TKI)) included in a phase II trial underwent CTC monitoring during treatment (*n*=41) [105]. A decrease in CTCs during treatment was associated with a tumor response, as evaluated by CT (*p*=0.019) or PET (*p*=0.014). For patients with *EGFR*-mutated NSCLC treated with the EGFR TKI, the CTC count can be followed during treatment (as done for ctDNA) levels) to monitor the treatment response [106, 107].

The use of specific protein expression on CTCs as predictive biomarkers is an interesting possibility. For cytotoxic chemotherapy in NSCLC patients, low excision repair cross-complementation group 1 (ERCC1) expression is associated with platinum sensitivity [108, 109]. Das et al. measured ERCC1 expression by immunohistochemical (IHC) analysis of CTCs from 17 patients with advanced NSCLC treated with platinum-based chemotherapy and found similar results: low ERCC1 expression on CTCs correlated with longer PFS [110]. Similarly, thymidylate synthase expression (associated with pemetrexed sensitivity in tumor samples) on CTCs has the same predictive effect [111]. Concerning immune checkpoints inhibitor (ICI) treatment, PDL1 expression (evaluated by IHC analysis) on tumor samples seems to correlate with the tumor response. Nicolazzo et al. performed serial IHC monitoring of PDL1 expression on CTCs from 24 NSCLC patients treated with nivolumab [112]. The persistence of PDL1-positive CTCs at 6 months was associated with a lack of clinical response to this treatment. Adams et al. showed also the feasibility of tracking the increase of PDL1-positive CTCs during radiotherapy in 100% of patients (n=41), whereas only 24% of primary biopsies had sufficient tissue for PD-L1 testing [113]. Therefore, the expression of predictive biomarkers on CTCs seems to be feasible, and could be a good surrogate to the use of IHC on small lung biopsies.

### CTCs as a diagnostic/screening tool

As described in several studies, CTCs are frequently detected in NSCLC patients, even at early disease stages, whereas the detection rate is null in healthy individuals. A small meta-analysis of five studies including control groups (total of 460 lung cancer patients and 239 controls with benign disease) showed that CTCs represent an efficient diagnostic tool, with a sensitivity of 75%, a specificity of 92%, and an area under the ROC curve (AUC) of 0.93 [114]. The presence of CTCs could be used for better characterization of lung nodules. Carlsson et al. studied 80 patients with stage I NSCLC and 25 patients with benign disease who underwent PET [115]. Detection of CTMs (by immunofluorescence assay) in addition to clinical and PET data was better for diagnosing lung cancer compared with clinical and PET only: AUC of 0.87 versus 0.77, respectively.

CTCs can be detected in patients with small lung tumors, and the question of using them as a lung cancer screening tool was partially answered by Ilie et al. [116]. These researchers investigated CTCs by ISET at baseline in 245 subjects without cancer: 168 patients with chronic obstructive pulmonary disease (COPD), 42 control smokers, and 35 nonsmokers. COPD patients underwent low-dose CT once a year for 5 years; they were heavy smokers (median 56 packet-years), and 49% had moderate to severe COPD. CTCs were detected in five COPD patients (3%) but no control patients. All five COPD patients developed NSCLC during the 5-year follow-up, as detected by CT (median time to node detection 3.2 years). None of the control patients developed a lung node during follow-up. Of the five NSCLC tumors, most were adenocarcinomas (*n*=4) and one was a squamous cell carcinoma. All were treated by surgery, which confirmed that all were stage Ia NSCLCs. However, these exciting results need to be prospectively confirmed in a larger population and the impact on reducing cancer-specific mortality remains a key determinant in validating this tool as an effective screening marker. It also surprising that none of the control patient developed a lung nodule, whereas around 25% of new nodules were found on CT-scan each year in smokers in a large screening clinical trial [117]. Moreover, the ISET technique still has some inter-user variation issues that limit its development for a use in routine so far.

### Clinical implication of CTCs in small cell lung carcinoma patients

SCLC is an aggressive lung cancer with high metastatic potential. The interest in monitoring CTC numbers in patients with this disease is therefore justified. As for NSCLC patients, CTCs can be detected and isolated in SCLC patients. Bevilacqua et al. isolated CTCs from four patients with extended SCLC for the first time using the Cellsearch system [118]. These results were confirmed in a cohort of 50 SCLC patients and 85 healthy controls [119]. The CTC detection rate was 86% using the Cellsearch system. The CTC count was a prognostic indicator: patients with >300 CTCs had worse survival than those with <2 CTCs (134 days versus 443 days, *p*<0.005). The same group confirmed this sensitivity level and the prognostic impact of CTC numbers in another cohort of SCLC patients (*n*=97) [120]. A multivariate analysis showed that the presence of CTCs at baseline was negatively associated with PFS (HR 2.01; 95% CI 1.17–3.46) and OS (HR 2.45; 95% CI 1.39–4.30), as was their presence after one cycle of chemotherapy (PFS: HR 4.20; 95% CI 1.44–12.25; OS: HR 5.49; 95% CI 1.78–16.91). The change in CTC numbers during chemotherapy was also associated with OS (HR 4.10; 95% CI 1.10–15.10). Similarly, Messaritakis et al. showed, using the Cellsearch system, in 108 SCLC patients, that chemotherapy reduced both the incidence of detection and the absolute number of CTCs, and that the incidence of detection and the number of CTCs were increased at the time of progression. Multivariate analyses confirmed the prognostic impact on OS of CTCs [121]. Several other studies reported similar results [122–129]. A meta-analysis (seven studies, 440 patients) on the prognostic impact of CTCs in SCLC patients found a HR for OS of 1.90 (95% CI 1.19–3.04) and for PFS of 2.60 (95% CI 1.90–3.54) [130].

An interesting approach to using CTCs in SCLC patients is the possibility of testing the chemosensitivity of isolated CTCs as for a reflect of tumor behavior in the patient. Hamilton et al. established two CTC lines from patients with extended SCLC [127] and tested these *in vitro* for chemosensitivity to topotecan and epirubicin [131]. Hodgkinson et al. implanted CTC explants isolated with the Cellsearch system into immunocompromised mice [132]. These explants had the same sensitivities to platinum and etoposide chemotherapy as the corresponding donor tumor. In comparison, CTCs explants in NSCLC are much more difficult to realize [133]. These possibilities of *in vivo* culture and CTCs explants in SCLC, associated with the high rate of CTCs isolated in SCLC patients, strengthen the interest of CTCs in this setting.

## MOLECULAR ANALYSES OF CTCS

For several years, molecular characterization has enabled the specific targeting of key oncogenic alterations (i.e. oncogenic addiction) in patients with advanced NSCLC with specific TKIs. [134–137]. However, the small size of tumor samples from NSCLC patients can make molecular screening a challenge in daily practice. Moreover, the need for a second round of molecular screening at the time of progression to identify resistance means that new biopsies are necessary during follow-up. The identification of somatic mutations in ctDNA isolated from blood samples (liquid biopsy) is a validated method of identifying *EGFR* mutations. However, detection of genetic rearrangements (such as *ALK*) in ctDNA is technically difficult. Molecular analysis of oncogenic addiction in CTCs therefore has huge potential for fully characterizing NCSLCs at baseline but also during the treatment of NSCLC patients with oncogenic addiction.

### EGFR mutations

In 2008, Maheswaran et al. reported the feasibility and sensitivity of detecting *EGFR* mutations in CTCs [138]. These researchers analyzed CTCs isolated by CTC-chip from 27 NSCLC patients. *EGFR* mutations were screened in ctDNA and CTCs using the Scorpion amplification refractory mutation system (SARMS) and in tumor samples using SARMS or standard sequencing. Of 20 patients with *EGFR*-mutated NSCLCs, the *EGFR* mutation was identified in 95% of the corresponding CTCs (*n*=19/20). Other studies confirmed these results using different molecular techniques, such as RT-PCR and melting curve analysis (sensitivity 100%, *n*=8) [139], next-generation sequencing (sensitivity 84%, *n*=37) [140], and SARMS (sensitivity 50%, *n*=4) [141]. Moreover, *EGFR* mutation can be easily detected in single CTCs [139, 142, 143].

In addition to detecting sensitizing *EGFR* mutations at diagnosis, longitudinal molecular screening of CTCs can also detect the emergence of resistance mutations, such as the exon 20 T790M *EGFR* mutation. Maheswaran et al. detected the T790M *EGFR* mutation at baseline in CTCs from 9 out of 14 patients who had disease progression after treatment with the EGFR TKI (64%) [138]. Serial molecular screening of CTCs was feasible, with the appearance and increasing prevalence of the T790M *EGFR* allele during gefitinib treatment (*n*=4). In seven advanced NSCLC patients who developed EGFR TKI resistance, Yeo et al. performed molecular screening of both tumor (re-biopsy) and CTC samples by direct sequencing [143]. The T790M *EGFR* mutation was detected in CTCs in four patients (57%) and in tumor samples from five patients (71% (one patient did not have detectable CTCs). The concordance rate for detection of the T790M *EGFR* mutation between CTC and tumor sample was good (κ=0.70). A larger study of 40 *EGFR*-mutated advanced NSCLC patients compared the incidence of the T790M *EGFR* mutation in tumor samples (*n*=40), CTCs (isolated by CTC-chip) (*n*=28), and ctDNA (*n*=32) [144]. All patients had disease progression after EGFR TKI treatment. The T790M *EGFR* mutation rate was 63% in tumor biopsies, 50% in CTCs, and 50% in ctDNA. Agreement between the different techniques was low to moderate: CTCs versus biopsy, κ=0.49; ctDNA versus biopsy, κ=0.29; and CTCs/ctDNA versus biopsy, κ=0.35. Notably, molecular screening of CTCs and ctDNA identified the T790M *EGFR* mutation in 35% of patients (*n*=14) with negative or indeterminate tumor sample findings. This discordance could be explained by technical issues, but also by tumor heterogeneity because CTCs and ctDNA represent all tumor clones whereas tumor biopsy represents a single site, as previously described [145, 146]. A meta-analysis in 2016 of *EGFR* mutation detection in CTCs (eight studies, 170 patients) found good sensitivity (91%) and specificity (99%), and an AUC of 0.99 [147].

However, despite this excellent performance, the role of *EGFR* screening in CTCs is still to be defined. The role of ctDNA is currently growing, notably for detecting the T790M *EGFR* mutation to monitor disease progression with EGFR TKI treatment. ctDNA analysis has fewer technical steps compared with CTC analysis, with similar performance [145, 146].

### ALK and ROS1 rearrangements

Detection of *ALK/ROS1* rearrangements is currently based on IHC or fluorescence *in situ* hybridization (FISH) in NSCLC tumor samples. Several studies have shown that these tests are feasible in CTCs from NSCLC patients. Ilie et al. performed *ALK* FISH and IHC analyses of CTCs (isolated by ISET) from 87 NSCLC patients [148]. There was perfect concordance for *ALK* status between CTC and tumor samples: *ALK* rearrangement was detected in CTCs and tumor samples in five patients (6%), whereas both CTC and tumor samples were negative in the remaining 82 patients. Several larger series of ALK-positive patients have confirmed the excellent performance of *ALK* FISH in CTCs [149–151]. Moreover, as shown with other anti-tumor treatments, the pattern of change in CTC count seems to correlate with the tumor response to ALK TKI [149–152]. Some assays have been developed for the simultaneous isolation and molecular analysis of CTCs by FISH. As an example, Pailler et al. developed a semi-automated microscopy method to identify filtration-enriched CTCs (ISET) by combining phenotypic and cytomorphological analysis with filter-adapted FISH, which enabled detection of *ALK-*rearranged CTCs in 82% of patients with *ALK*-rearranged NSCLC [153].

As with EGFR TKI treatment, it is also feasible to perform serial molecular screening of CTCs during treatment of *ALK*-rearranged NSCLC patients. Zhang et al. described a NSCLC patient with an *ALK* translocation identified in both the tumor sample and CTCs (by FISH) and treated with crizotinib [154]. At the time of disease progression, an *ALK* resistance mutation was detected in both a new tumor biopsy (L1196M) by whole exome sequencing and in CTCs isolated at the same time using Sanger sequencing. The patient then received treatment with a second-generation ALK TKI (ceritinib).

similar to *ALK* rearrangement, *ROS1* rearrangement can also be detected in CTCs by filter-adapted FISH [155].

### Other molecular abnormalities

*Kras* mutation is the most frequent molecular abnormality in NSCLC patients, occurring in around 25% of tumors [2]. This mutation can be detected in both ctDNA and CTCs. However, several studies have highlighted that *Kras* mutation detection in CTCs is less sensitive compared with ctDNA (e.g. using high-sensitive assays such as digital droplet PCR (ddPCR)) [156, 157]. Similar results for other mutations, e.g. *Braf* [158], question the place of CTC analysis in point mutation screening.

### Extensive molecular screening of CTCs

Compared with screening for one molecular abnormality, large-scale screening of multiple abnormalities is more challenging but represents a promising tool for developing new targeted therapies. The rapid development of new assays for extensive gene profiling, most commonly in single CTCs, provides further applications of CTCs in NSCLC patients. Park et al. performed multigene (four-gene) expression and mutation profiling of single CTCs from 35 stage IV lung adenocarcinoma patients, and reported high sensitivity [159]. Larger panels of cancer-associated genes can also be used. For example, Yoo et al. reported the feasibility of large molecular screening with a panel of 381 cancer-related genes in single CTCs, confirmed by ddPCR. In a cohort of 13 NSCLC patients, the detection rate of point mutations was 62% (*n*=8) in CTCs and 85% (*n*=11) in tumor samples, mainly in *EGFR*, *TP53*, and *FGFR* genes. Interestingly, some mutations detected in CTCs were not observed in the corresponding tumor samples, suggesting that CTCs can have a distinct genetic profile. Copy-number alterations (CNAs) can also be evaluated in CTCs [160–162]. Carter et al. evaluated CNAs in CTCs from two SCLC cohorts (training set, *n*=13 patients; validation set, *n*=18 patients) [162]. These researchers were able to classify 83% of tumors as chemosensitive or chemorefractory based on the CNAs profile. Moreover, the CNA-based classification had a prognostic impact on PFS. Extensive exome analysis by whole exome sequencing is also possible in single CTCs. The inherent limitation of low-quantity of DNA extracted from single CTCs can be partly resolved by whole exome amplification, which enables sufficient DNA to be produced to perform further deep genome analyses [163]. However, DNA quality affected by the CTC isolation process, cell fixation, DNA amplification, and the low starting DNA quantity remain potential issues [163]. Last, whole transcriptome analysis (WTA) enables global gene expression in CTCs to be evaluated and key regulators of lung cancer cells to be identified. For example, WTA analysis of EpCAM-positive CTCs from 42 NSCLC patients showed that the most highly expressed genes in CTCs are linked to cell movement, cell adhesion, and cell–cell communication, such as Notch1, and have high prognostic value [164].

## LIMITATIONS AND PERSPECTIVES

Despite the exciting results on CTCs in lung cancer, the applicability of a clinical use of CTCs in the management of NSCLC is still subject to discussion. Beyond the wide diversity of available assays, each one with potential limitation (Table 1), the main problem is still to have a consistent definition of CTCs. One major question is whether CTCs really represent the tumor, or if they are only particular cancer cells, with EMT and/or CSC features. As detailed above with the multiple techniques developed for CTCs isolation, there is currently no universal definition of CTCs. Depending on the device, CTCs will be EpCAM-expressing cells, CD45-negative cells, and/or large circulating cells. This question is a major limitation for comparison of results in the literature, integration in clinical trials in oncology and for generalization of CTCs use in the daily practice. Consortia such as Cancer-ID are aiming to standardize these aspects. Moreover, it is important to note that, until now, CTCs use has never been shown to prolong patient survival yet. A prospective randomized trial using CTCs to manage advanced NSCLC treatment is still awaited, but should use validated assay for CTCs isolation, strict time-points for CTCs analyses and meaningful thresholds, as these parameters can largely influence the results of the trial, as shown in other malignancies [165, 166]. Current recruiting phase I-III clinical trials using CTCs in lung cancer are presented in Table 2.

**Table 1:** Main assays developed for CTCs detection and isolation

CTCs detection/isolation assay	Sensitivity	Specificity	Limitations	Ref
EpCAM-based assay (Cellsearch)	20-100%	80-95%	no EMT CTCs detection	[10–16]
CD45- based assay	73-100%	82%	limited number of studies	[17–18]
Chip-based assay (CTC-chip…)	36-99%	100%	purity sometimes low	[23–43]
Size-based assay (ISET, ScreenCell, MCA…)	41-80%	90-100%	poor inter-institution correlation rate (ISET)	[44–54]

**Table 2:** Recruiting phase I-III clinical trials using CTCs in lung cancer

Type of trial	Study title	ClinicalTrials.gov Identifier
Phase II	Liquid Biopsy as a Tool to Evaluate Resistance to First and Third (AZD9291) (EGFR) (TKIs) in (EGFR) Mutant NSCLC	NCT02771314
Phase I-II	High-activity Natural Killer Immunotherapy for Small Metastases of Non-small Cell Lung Cancer	NCT03007875
Phase I-II	Combination of Cryosurgery and NK Immunotherapy for Advanced Non-Small Cell Lung Cancer	NCT02843815
Phase I-II	Combination of Cetuximab and NK Immunotherapy for Recurrent Non-small Cell Lung Cancer	NCT02845856
Phase I-II	Safety and Efficiency of γδ T Cell Against Lung Cancer	NCT03183232
Phase II-III	Bioinformation Therapy for Lung Cancer	NCT03239171

**Figure 1:**
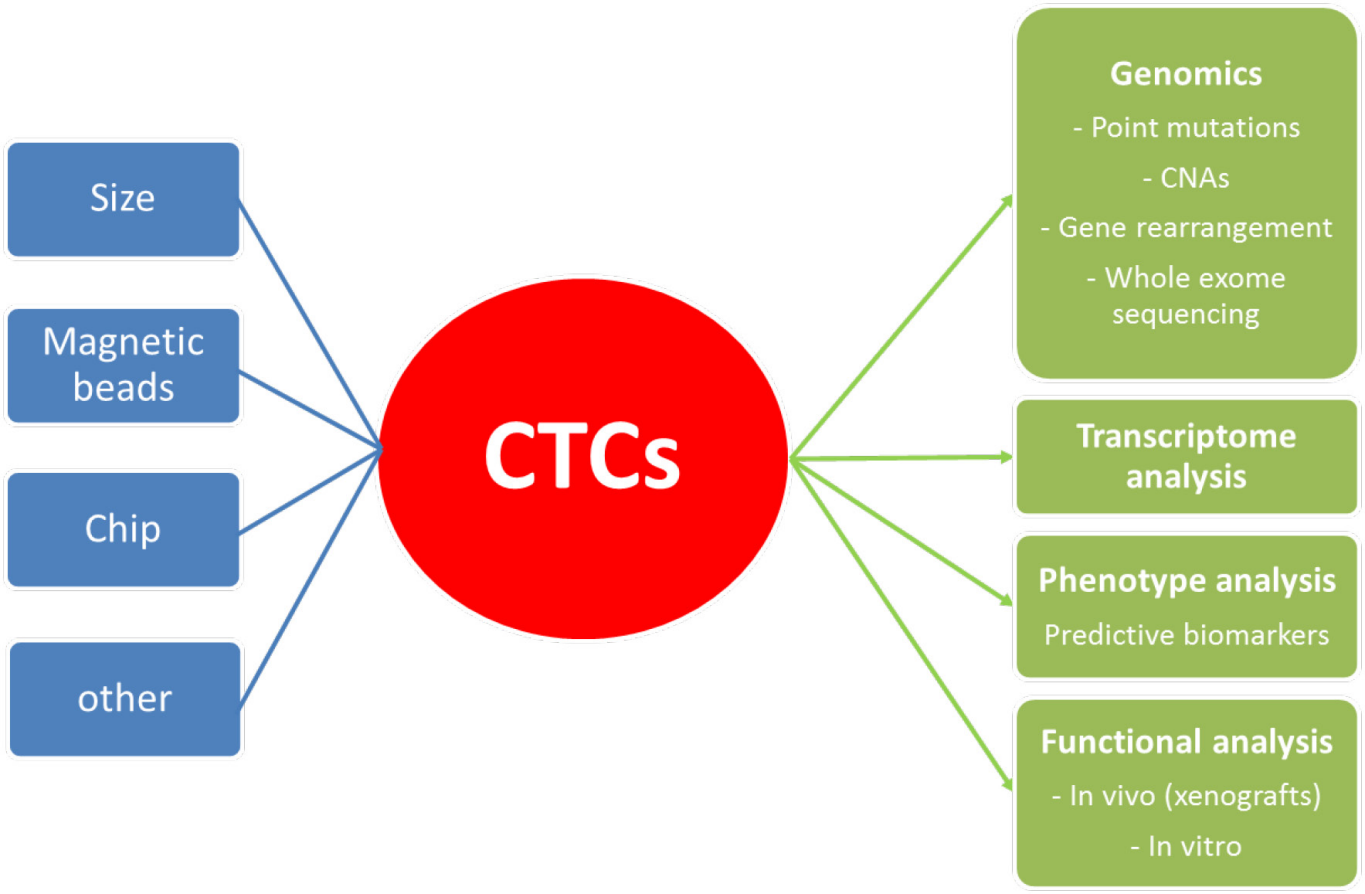
Methods of detection and molecular applications of CTCs CNAs: copy number alterations.

Another potential issue for the future development of CTCs in lung cancer is the putative advantage of CTCs among ctDNA. ctDNA is now widely used in lung cancer as a diagnostic, prognostic and predictive biomarker. Current techniques allow very low detection rate for somatic mutation in ctDNA, lower than 0.01% with ddPCR [3]. Large molecular screening by NGS is feasible on ctDNA, with good performances [167]. Based on these data, the detection of somatic mutations in lung cancer through analysis of CTCs does not seem to give a definitive advantage over ctDNA. Besides, it adds a supplemental technical step (CTCs isolation), that raises potential issue by itself, as discussed above, with additional costs. However, CTCs could have an interest in specific situations in addition with ctDNA. Interestingly, in the study of Sundaresan et al. on the T790M mutation detection (n=37), the mutation could be detected in CTCs and not in corresponding ctDNA (n=5), or in ctDNA and not in corresponding CTCs (n=1). Moreover, the use of both CTCs and ctDNA increased the T790M detection rate to 100%, and allowed the detection of the mutation in 35% patients s in whom the concurrent biopsy was indeterminate or T790M negative. CTCs offer also several advantages compared to ctDNA analysis (Figure 1). Notably, the possibility of transcriptome analyses (gene expression screening…) and biomarkers expression assessment (PDL1 for example) are real advantages of CTCs that cannot be performed with ctDNA. At last, the use of CTCs for functional analyses (chemosensitivity…) with tumor explants *in vivo*, especially in SCLC, is probably the most exciting perspective for CTCs use in the future.

In conclusion, the use of CTCs in lung cancer opens a wide range of applications that need to be validated for a clinical use, especially in comparison with other liquid biopsies (ctDNA). Moreover, the diversity of assays for CTCs detection and isolation, with several limitations for each technique, limits currently the application of CTCs in clinical research. A work of standardization concerning the definition of CTCs, the way to isolate them and their application in clinical trials is therefore needed.
